# *Aedes aegypti* exhibits a distinctive mode of late ovarian development

**DOI:** 10.1186/s12915-023-01511-7

**Published:** 2023-01-24

**Authors:** Heng Zhang, Feng Guang Goh, Lee Ching Ng, Chun Hong Chen, Yu Cai

**Affiliations:** 1 Temasek Life Sciences Laboratory, National University of Singapore, Singapore, 117604 Singapore; 2 Department of Biological Sciences, National University of Singapore, Singapore, 117543 Singapore; 3 Environmental Health Institute, National Environment Agency, 11 Biopolis Way, #06-05/08, Helios Block, Singapore, 138667 Singapore; 4 National Institute of Infectious Diseases and Vaccinology, National Health Research Institutes, Zhunan, Miaoli 350401 Taiwan

**Keywords:** *Aedes aegypti*, *Drosophila melanogaster*, Ovariole formation, Terminal filament, Cyst-like proliferation, TOR signaling, Ecdysone, *Culex quinquefasciatus*, *Anopheles sinensis*, Diptera

## Abstract

**Background:**

Insects live in almost every habitat on earth. To adapt to their diverse environments, insects have developed a myriad of different strategies for reproduction reflected in diverse anatomical and behavioral features that the reproductive systems of females exhibit. Yet, ovarian development remains largely uncharacterized in most species except *Drosophila melanogaster* (*D. melanogaster*), a high Diptera model. In this study, we investigated the detailed developmental process of the ovary in *Aedes aegypti* (*Ae. aegypti*), a major vector of various disease-causing pathogens that inhabits tropical and subtropical regions.

**Results:**

Compared with *Drosophila melanogaster*, a model of higher Diptera, the processes of pole cell formation and gonad establishment during embryonic stage are highly conserved in *Ae. aegypti*. However, *Ae. aegypti* utilizes a distinct strategy to form functional ovaries during larval/pupal development. First, during larval stage, *Ae. aegypti* primordial germ cells (PGCs) undergo a cyst-like proliferation with synchronized divisions and incomplete cytokinesis, leading to the formation of one tightly packed “PGC mass” containing several interconnected cysts, different from *D. melanogaster* PGCs that divide individually. This cyst-like proliferation is regulated by the target of rapamycin (TOR) pathway upon nutritional status. Second, ecdysone-triggered ovariole formation during metamorphosis exhibits distinct events, including “PGC mass” breakdown, terminal filament cell degeneration, and pre-ovariole migration. These unique developmental features might explain the structural and behavioral differences between *Aedes* and *Drosophila* ovaries. Importantly, both cyst-like proliferation and distinct ovariole formation are also observed in *Culex quinquefasciatus* and *Anopheles sinensis*, suggesting a conserved mode of ovarian development among mosquito species. In comparison with *Drosophila*, the ovarian development in *Aedes* and other mosquitoes might represent a primitive mode in the lower Diptera.

**Conclusions:**

Our study reveals a new mode of ovarian development in mosquitoes, providing insights into a better understanding of the reproductive system and evolutionary relationship among insects.

**Supplementary Information:**

The online version contains supplementary material available at 10.1186/s12915-023-01511-7.

## Background

Insects represent the most diverse organisms in the world, living in almost every habitat on earth and prevailing in various environments. Females develop a myriad of strategies for reproduction which include the formation of diverse ovarian structures and the establishment of reproductive behaviors adapted to their residential environments [1]. Ovarioles, egg-producing units of insect ovaries, can be categorized into two main types: panoistic and meroistic [2]. In panoistic ovarioles, oocytes arise from the mitotic divisions of oogonial cells, with a complete cytokinesis. In contrast, in meroistic ovarioles, the incomplete cytokinesis of oogonial cell divisions results in interconnected germ cell cysts. Meroistic ovariole contains two subtypes: telotrophic meroistic ovarioles with nurse cells residing in the germarium and polytrophic meroistic ovarioles with nurse cells and oocyte co-develop into follicles. Although morphological diversity exists, it is generally documented that an insect ovariole consists of two regions: an anterior germarium and a posterior vitellarium [1]. The germarium usually contains oogonial cells and their associated stromal cells, with a terminal filament (TF) attached anteriorly, whereas the vitellarium consists of a series of developing oocytes, often with a pedicel or ovariole stalk attached posteriorly.

Among insect species, the development of female reproductive system of *D. melanogaster* in Diptera is best studied [3]. *D. melanogaster* ovarian development begins with the formation of primordial germ cells (PGCs) specified by maternally deposited germ plasm during early embryogenesis [4, 5]. Subsequently, PGCs migrate from extra-embryonic position into the gonadal region, split, and coalesce with gonadal somatic cells to form two gonads [6]. During the early larval stages, PGCs are enclosed by somatic intermingled cells (ICs) and divide independently to expand their numbers [7]. During the late larval stage, TF cells are specified among the apical somatic cells to form TF stacks, which serve as a singling hub to recruit other somatic precursors and PGCs, forming ovarioles during larval/pupal transition [8]. During this process, TF orchestrates the formation of germline stem cell (GSC) niche, while those somatic cells at the basal side of the ovary form an ovariole stalk. In adult, each ovary consists of about 17 polytrophic meroistic ovarioles, whereas each ovariole contains a germarium followed by 6–7 developing follicles (each with 15 nurse cells and 1 oocyte), which support continuous oogenesis [3].

*Ae. aegypti* mosquito is a hematophagous insect belonging to Diptera, with a different life cycle and reproduction strategy. The female mosquito contains a pair of ovaries, with each ovary harboring about 100 polytrophic meroistic ovarioles. Each ovariole contains a germarium followed by one to two follicles (each with 7 nurse cells and 1 oocyte), with the primary follicles arrested at the previtellogenic (resting) stage [9]. Upon a successful blood meal, the primary follicles develop synchronously into mature eggs within a few days. Meanwhile, the secondary follicles develop into the previtellogenic resting stage, followed by a new batch of follicles that emerge from the germaria. This ovarian cycle is under the concerted regulation of endocrine hormones, including juvenile hormone, ecdyseteriod, and grow factors like insulin [9–13]. To some extent, the germline development of *Ae. aegypti* shares conservative features with that of *D. melanogaster*, particularly during the embryonic stage. In their histological study, Raminani and Cupp documented that *Ae. aegypti* (Florida strain) germline development during early embryogenesis is initiated with the formation of 14–16 pole cells, which migrate and split to form two gonadal rudiments, each containing 4–6 pole cells, similar to that of *D. melanogaster* [14, 15]. Apart from these, the detailed process of ovarian development of *Ae. aegypti*, especially during the larval and pupal stages, remains largely uncharacterized. *Ae. aegypti* is a major vector of various disease-causing arboviruses, including dengue and Zika viruses. Notably, many vector control methods, including sterile insect technique (SIT), *Wolbachia*-based incompatible insect technique (IIT), and gene drive systems, target mosquito’s female reproductive systems [16–21]. Hence, understanding the germline development of *Ae. aegypti* might lead to the development of new effective population-based vector control method(s).

In this study, we showed that although the embryonic ovarian development of *Ae. aegypti* is similar to that of *D. melanogaster*, *Ae. aegypti* undergoes different processes to expand its PGC pools and form functional ovarioles. During the larval stage, *Ae. aegypti* PGCs undergo cyst-like synchronized divisions with incomplete cytokinesis, forming one tightly packed “PGC mass” that contains several PGC cysts without somatic ICs. This is different from *D. melanogaster* PGCs, which are wrapped by somatic ICs and proliferate asynchronously. The cyst-like proliferation of PGCs in *Ae. aegypti* is likely controlled by the TOR pathway in response to environmental nutrition, which is closely related to ovarian size and fecundity at the adult stage. Another key feature in *Ae. aegypti* is the unique process of ovariole formation. Transitory TF stacks form upon ecdysone to initiate ovariole formation during larval/pupal transition but degenerates at the late pupal stage. Consequently, *Ae. aegypti* adult ovariole lacks TF, which is distinct from *D. melanogaster* ovariole in which TF acts as a niche component at the adult stage. During metamorphosis, the ovary of *Ae. aegypti* undergoes a massive morphogenetic movement. Ovarioles migrate from one side of the ovary to cover its entire surface. Meanwhile, the ovarioles reorientate from the lateral to the anterior/posterior (A/P) axis, with basal ovariole stalks pointing posteriorly. Importantly, the cyst-like proliferation of PGCs and those unique features that occur during ovariole formation were also observed in other mosquito species, including *Culex quinquefasciatus* and *Anopheles sinensis*, suggesting a conserved mode of ovarian development in mosquitoes. Our results thus reveal an alternative mode of ovarian development in insects.

## Results

### Embryonic gonadal development is conserved between *Ae. Aegypti *and* D. melanogaster*

**Fig. 1:**
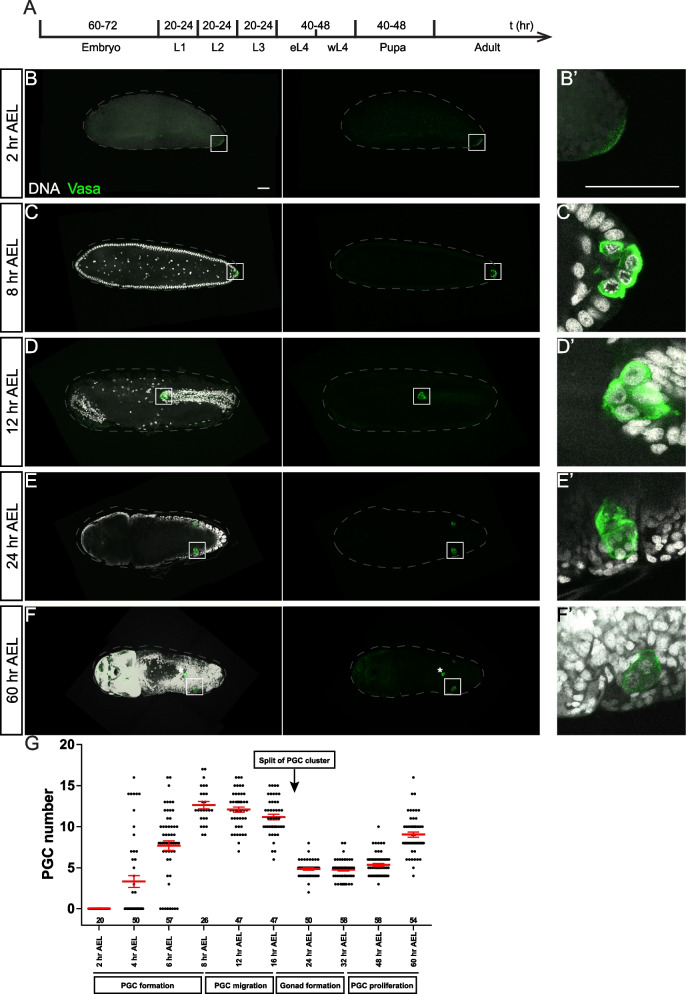
Germline development during the embryonic stage is highly conserved. **A** Time course of *Ae. aegypti* developmental stages under standard laboratory condition. Confocal images of embryos (**B**–**F**) and zoomed-in for the outlined area (**B’**–**F’**) at 2 h AEL (**B**, **B’**), 8 h AEL (**C**, **C’**), 12 h AEL (**D**, **D’**), 24 h AEL (**E**, **E’**), and 60 h AEL (**F**, **F’**) with Vasa (green) and DNA (white) staining. **B**, **B’** Vasa crescent detected at the posterior end of the embryo. **C**, **C’** PGCs formed at the posterior end of the embryo. **D**, **D’** PGCs detected on migrating germ band. **E**, **E’** PGCs detected in two gonads. **F**, **F’** PGCs in two gonads at 60 h AEL. **G** Quantification of PGC number in the embryo (before gonad formation, 0–16 h AEL) or in each gonad (after gonad formation, 24–60 h AEL). Data are represented as mean ± SEM. The number of samples (*n*) in each group is shown above the *X*-axis. The dash lines mark the outlines of embryos. The asterisk in **F** marks the mismigrated PGC. Scale bars in **B** and **B’** are 50 µm

Under the standard breeding condition (see the “Methods” section), embryogenesis took about 60 to 72 h in *Ae. aegypti* (NEA-EHI strain) (Fig. 1A). In order to label PGCs during germline development, we generated antibodies against *Ae. aegypti* Vasa (AaegVasa or Vasa herein, AAEL004978), a conserved germ cell marker across metazoan [22]. Between 0 and 2 h after egg laying (AEL), embryos contained a few interiorly localized nuclei. During this stage, Vasa was detected as a crescent located at the posterior end of the embryos (Fig. 1B, B’), similar to that observed in *D. melanogaster* [23, 24]. Pole cell formation was observed at 4 h AEL, with pole buds detected (Fig. 1G and Additional file 1: Fig. S1A-S1A”). Following that, the PGC number increased from 4 h (3.3 ± 0.7, *n* = 50) to 8 h AEL (12.6 ± 0.4, *n* = 26) (Fig. 1C, C’, G). PGCs were subsequently carried by surrounding tissues during germband extension. By 12 h AEL, a closely packed group of PGCs (12.1 ± 0.3, *n* = 47) was observed at the tip of the extending germband (Fig. 1D, D’, G). After reaching the midgut pocket within the invaginated embryo, PGCs underwent a trans-midgut migration into the interior of the embryo. PGCs then split into two groups, migrated, and eventually coalesced with gonadal somatic cells to form two embryonic gonads (Additional file 1: Fig. S1B-B”). Some PGCs failed to migrate properly during this process (Fig. 1F and Additional file 1: Fig. S1C-S1D’), and similar observations were previously reported during *D. melanogaster* PGC migration [25, 26]. By 24 h AEL, each gonad located on the dorsal side of the retracting germband contained an average of 4.8 ± 0.1 PGCs/gonad (*n* = 50) (Fig. 1E, E’, G). By the completion of gonad formation, about 24% of PGCs failed to reach embryonic gonads. PGC numbers increased slightly and reached 5.3 ± 0.2 PGCs/gonad (*n* = 58) by 48 h AEL (Fig. 1G). The proliferation of PGCs in each gonad was observed from 48 h AEL (5.3 ± 0.2/gonad, *n* = 58) and reached an average of 9.0 ± 0.3 PGCs/gonad (*n* = 54) by 60 h AEL, shortly before the completion of embryogenesis (Fig. 1F, G). Collectively, embryonic germline development in *Ae. aegypti* shares many features with that of *D. melanogaster*, including PGC formation, migration, gonad formation, and limited PGC proliferation [6].

### PGC number continues to increase during larval development

From L1 to L3 stages, each larval stage took about 20 to 24 h, while L4, which is arbitrarily divided into early L4 (eL4) and wandering L4 (wL4), took about 40 to 48 h (Fig. 1A).

**Fig. 2:**
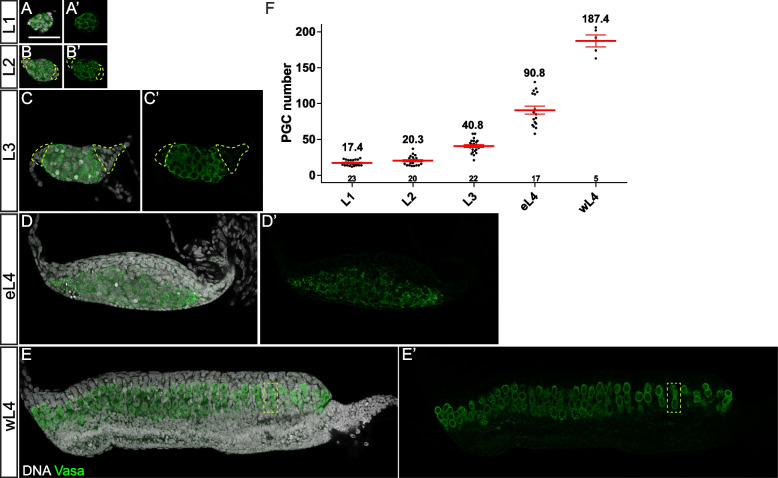
PGC mass increases during the larval stage. **A**, **A’**, **B**, **B’**, **C**, **C’**, **D**, **D’**, **E**, **E’** Confocal images of the germline during L1 (**A**), L2 (**B**), L3 (**C**), eL4 (**D**), and wL4 (**E**) stages with Vasa (green) and DNA (white) staining. **A**, **A’**, **B**, **B’**, **C**, **C’**, **D**, **D’** PGCs continue to increase and form PGC mass from L1 to eL4. **E**, **E’** PGC mass breaks into smaller clusters (one cluster indicated by a yellow rectangle) during the wL4 stage. **F** Quantification of PGC number from L1 to wL4 stages. Data are represented as mean ± SEM. The number of samples (*n*) in each group is shown above the *X*-axis. See Additional file 2 for the detail of counting information. Yellow dashed lines in **B**, **B’**, **C**, and **C’** mark somatic cells on both ends. Scale bar in **A** is 50 µm

Shortly after hatching, the number of larval PGCs continued to increase, similar to that observed in *D. melanogaster* [1]. PGCs increased rapidly with a doubling time of around 20 h, resulting in an average of 17.4 ± 0.8 (*n* = 23), 20.3 ± 1.5 (*n* = 20), 40.8 ± 2.0 (*n* = 22), 90.8 ± 5.6 (*n* = 17), and 187.4 ± 8.2 (*n* = 5) PGCs at L1, L2, L3, eL4, and wL4, respectively (Fig. 2F and Additional file 2: Table S1).

During L1, we were not able to distinguish the ovary from the testis by morphological features. Thus, the PGC number was reflected by the average number of all L1 gonads examined (Fig. 2A, A’, F), while from L2 onwards, the ovaries showed distinct morphologies from the testes. First, the ovaries were oval-shaped while the testes were rounder in shape (Fig. 2B, B’ and Additional file 3: Fig. S2). Second, the ovaries but not the testes contained multiple layers of somatic cells at both ends, a structure similar to that of L3 ovary shown in Fig. 2B, B’, C, C’ and Additional file 3: Fig. S2 (marked by yellow dashed lines). From L2 to eL4, the PGC number further increased within a tight cluster, which is referred to as “PGC mass” (Fig. 2B, B’, C, C’, D, D’, F). Meanwhile, gonadal somatic cells increased, and the ovary grew along the A/P axis with an elongated shape (Fig. 2B–D). During the wL4 stage, a rapid increase of somatic cells was observed, and the ovary was further elongated. Meanwhile, the large “PGC mass” was reorganized to form a long stretch with a width of about 3 PGCs before breaking down into small groups containing 2–6 PGCs. Some somatic cells were subsequently observed between these small PGC groups and surrounded them to form separate units, referred to as “pre-ovarioles” (Fig. 2E, E’, marked by a yellow rectangle, also refer to the “Ovariole is formed via a distinct mode during metamorphosis in *Ae. aegypti*” section).

### PGCs undergo germline cyst-like proliferation during larval development

In *D. melanogaster*, PGCs are separated by somatic ICs, and each PGC has its own cell cycle program independently of other PGCs. The *D. melanogaster* ICs play an important role in controlling the proliferative activity of the enclosed PGCs [7, 27]. Notably, *Ae. aegypti* PGCs seem to form only one tightly packed PGC mass without observed IC-like cells during early larval stages (Fig. 2A, A’, B, B’, C, C’, D, D’). In order to investigate the structure of the PGC mass, we searched for additional germ cell markers and focused on the components of fusome, a germ cell-specific intracellular organelle observed in several insect orders [28]. Fusome is an endoplasmic reticulum (ER) extension enriched in Actin and membrane cytoskeletal proteins, including α-Spectrin [29]. In *D. melanogaster*, the fusome appears spherical in PGCs and GSCs, referred to as spectrosome, but transforms into an elongated and branched structure in germline cysts as a result of incomplete cytokenesis [30]. Thus, the morphology (spherical vs. branched) of the fusome can serve as an indicator of distinct developmental states (germline stem cell/progenitor vs. differentiating germline cyst) of germ cells. After screening available fusome markers from *D. melanogaster*, we found that phalloidin and anti-*Drosophila* α-Spectrin antibody label fusome of *Ae. aegypti* germ cells (Additional file 4: Fig. S3A-B) [28]. Their signals were largely overlapped with a subtle difference, thus providing a better visualization of fusome when both were applied. We thus used a double labeling of these two markers in the following experiments.

**Fig. 3:**
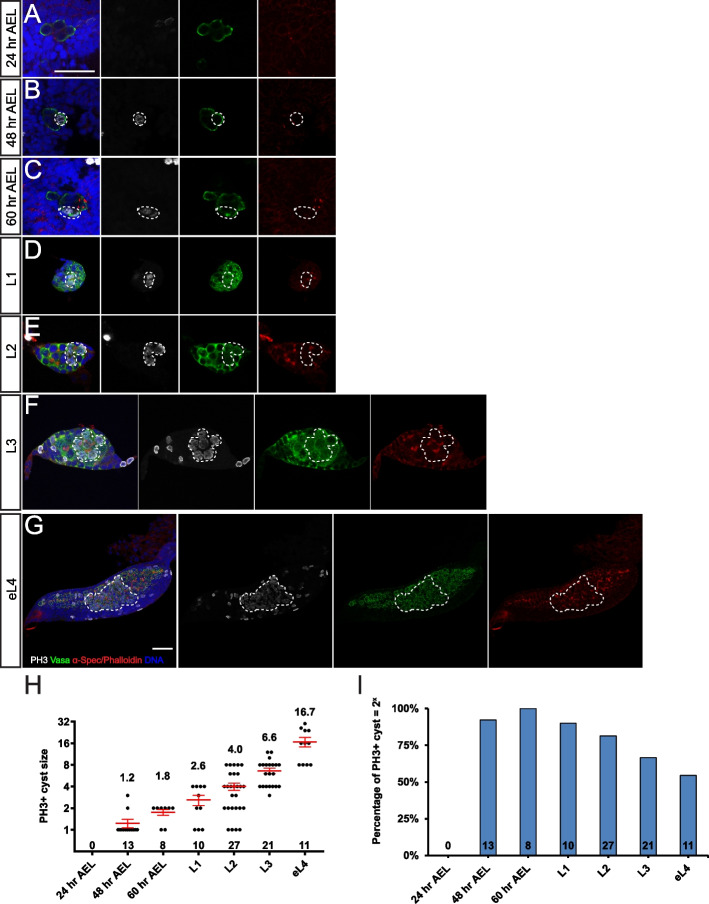
PGC proliferates as interconnected cysts. Confocal images of the germline at 24 h AEL (**A**), 48 h AEL (**B**), 60 h AEL (**C**), L1 (**D**), L2 (**E**), L3 (**F**), and eL4 (**G**) with Vasa (green), α-Spec/phalloidin (red), DNA (blue), and PH3 (white) staining. **A** The gonad shows no PH3-positive PGC at 24 h AEL. **B**–**G** PH3-positive PGCs (white) appear to form a cyst connected by fusome (red). **H** Quantification of PH3-positive cyst size from 24 h AEL to eL4 stages. Data are represented as mean ± SEM. **I** The percentage of PH3 + PGC cyst of a size that corresponds to a power of two. The number of samples (*n*) in each group is shown above the *X*-axis. The dash lines mark the PH3 + PGC cysts. Scale bar in **A** indicates 50 µm (for **A**–**F**). Scale bar in **G** indicates 50 µm

In *Ae. aegypti*, the fusomes appeared spherical in shape and were weakly detected during the embryonic stage (Fig. 3A–C). Unexpectedly, during the PGC proliferation period at the early larval stages, branched fusomes were observed (Fig. 3D–G), instead of spectrosomes which are normally detected in proliferating PGCs of *D. melanogaster* [31]. A careful examination showed that these fusomes run through the ring canals to connect PGCs within a cluster (Additional file 4: Fig. S3A). Through 3D reconstruction, we found that L1 and L2 ovaries contain 4–5 fusomes, each connecting a group of PGCs which we name as “PGC cyst” (Additional file 5: Movie S1 and Additional file 6: Movie S2). Of note, the number of fusomes is close to 4.8 PGCs in the newly formed embryonic gonad (Fig. 1G), suggesting that each PGC cyst is likely derived from a single PGC through incomplete cytokinesis. From the L3 stage onwards, some of the less branched fusomes were also observed, which might derive from the breakdown of highly branched fusomes (Fig. 3F, G). These data show that during the early larval stages, *Ae. aegypti* PGCs form a large PGC mass containing several interconnected PGC cysts, different from that observed in *D. melanogaster* PGCs, which divide as individual cells.

Another hallmark of interconnected germline cysts is the shared cytoplasm and synchronized cell cycle progression [32]. We next investigated whether these fusome-connected PGC cysts undergo a synchronized proliferation. We used anti-phospho-histone H3 (pSer10) or PH3 antibody, a mitotic marker, to directly investigate the mitotic synchrony of the PGC cysts. At 24 h AEL, no PH3-positive PGC was detected (Fig. 3A, H, I). Some single/two-cell PH3-positive PGCs were detected at 48 h and 60 h, consistent with the window of PGC proliferation (Figs. 1G, 3B, C, H). However, from the L1 stage onwards, most PH3-positive PGCs formed clusters (Fig. 3D–G). Co-labeling with fusome markers revealed that these mitotic PGCs belong to the same PGC cyst in a similar mitotic phase, supporting the notion of the mitotic synchrony (Fig. 3D–G and Additional file 6: Video S2). Of note, the size of PH3-positive PGC cysts increased concomitantly with germline development and frequently corresponded to a power of 2, a key feature of germline cyst division (Fig. 3H). For instance, L1 ovaries typically contained 2-cell or 4-cell clusters, and L2 ovaries contained 4-cell or 8-cell clusters, while 16-cell cysts were observed only in L4 ovaries. However, the portion of mitotic PGC cysts corresponding to a power of 2 reduced gradually from L1 to L4 (Fig. 3I), suggesting a PGC cyst breakdown during proliferation. This is consistent with previous studies on germline development in mice [33, 34].

Mitotic synchrony was further supported by EdU pulse-chase labeling experiment. By exposing L3 larvae to EdU-containing solution for a short period, EdU is incorporated into the chromatin undergoing DNA synthesis. It allows to label PGCs that are in the S phase but not those in the mitotic phase. Indeed, while the majority of PGCs were labeled by EdU, some PGC cysts were EdU-negative, indicating the mitotic synchrony during the pulse period (Additional file 4: Fig. S3C). Furthermore, shared cell fate within a PGC cyst was further supported by synchronized cell death triggered by acute starvation (Additional file 4: Fig. S3D).

Collectively, these data reveal that *Ae. aegypti* PGC mass consists of several interconnected PGC cysts, which undergo multiple rounds of synchronous divisions with incomplete cytokinesis during the larval stage. In contrast to *D. melanogaster* in which PGCs proliferate by dividing independently of each other [3], *Ae. aegypti* expands its PGC pool via a unique cyst-like proliferation manner.

### PGC cyst-like proliferation rapidly responds to nutritional status

**Fig. 4:**
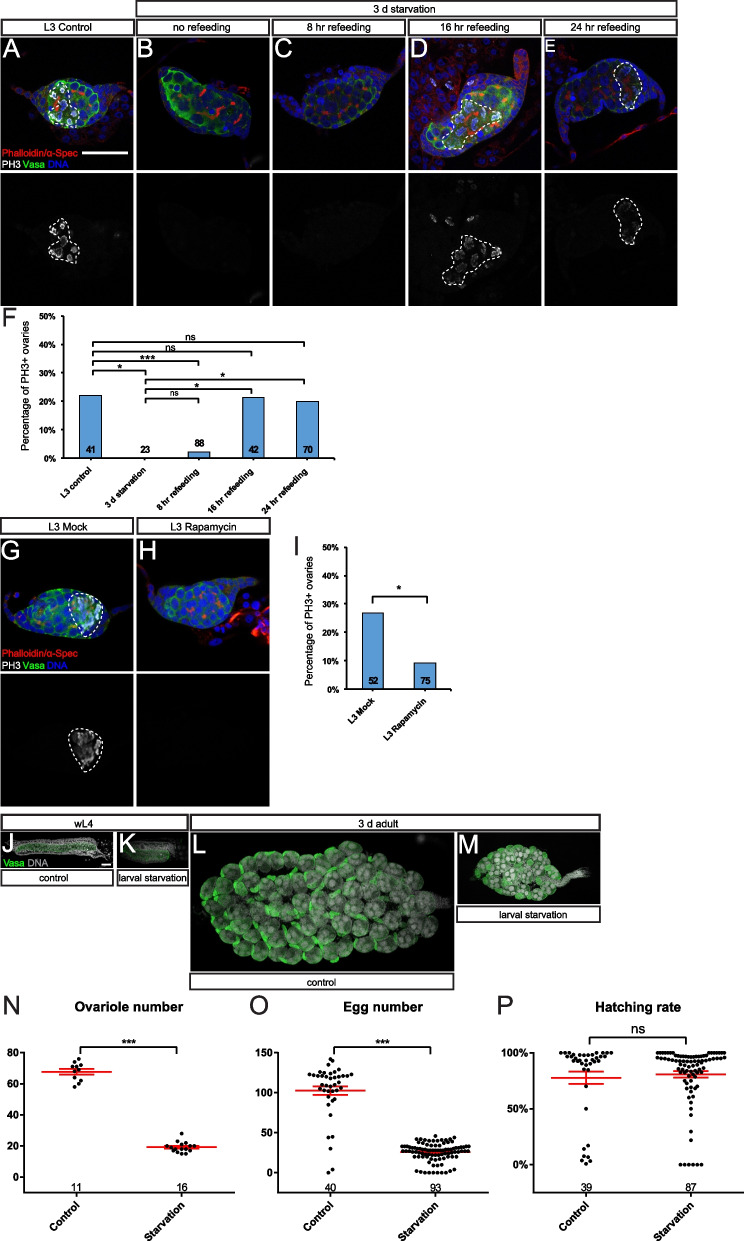
PGC cyst-like division responds to nutritional status promptly. Confocal images of L3 ovaries of control (**A**), starved for 3 days (**B**), 8 h after refeeding (**C**), 16 h after refeeding (**D**), or 24 h after refeeding (**E**) with Vasa (green), α-Sepc/phalloidin (red), DNA (blue), and PH3 (white) staining. **A** A well-fed control L3 ovary shows PH3 + PGC cyst. **B** A L3 ovary after 3-day starvation does not contain proliferating PGC cyst. **C** A L3 ovary after 8 h refeeding does not contain proliferating PGC cyst. Starved L3 ovary shows PH3 + PGC cyst at 16 h (**D**) and 24 h (**E**) after refeeding. **F** Quantification of percentages of ovaries with PH3-positive PGCs from three to five pooled independent replicates for **A**–**E** using Fisher’s exact test. Confocal images of mock-treated (**G**) and rapamycin-treated L3 ovaries for 16 h (**H**) with Vasa (green), α-Spec/phalloidin (red), DNA (blue), and PH3 (white) staining. **I** Quantification of percentages of ovaries with PH3-postive PGCs from three pooled independent replicates for **G** and **H** using Fisher’s exact test. Confocal images of ovaries of wL4 (**J**, **K**) and 3-day females (**L**, **M**) with Vasa (green) and DNA (white) staining. Compared to control (**J**, **L**), the size of the ovaries reduces dramatically under long-term starvation (**K**, **M**). **N**–**P** Quantifications of ovariole number, egg number, and hatch rate of females from control and long-term starved larvae from three pooled independent replicates using non-parametric *t*-test. Data are represented as mean ± SEM. The number of samples (*n*) in each group is shown above the *X*-axis. ***p < 0.001.*p < 0.05. ns, not significant. The dash lines mark PH3 + PGC cysts. Scale bar in **A** indicates 50 µm (for **A**–**E**, **G**, **H**). Scale bar in **J** indicates 50 µm (for **J**–**M**)

We next investigated the physiological significance of this PGC cyst-like proliferation during *Ae. aegypti* larval development. In the field, one of the most common environmental challenges is food availability, when mosquito larvae can survive with a growth arrest for days without food and resume to grow shortly after refeeding [35]. Considering that a cyst-like mitotic synchrony might be an efficient way to regulate PGC proliferation, we designed a starvation/refeeding regime to examine the dynamics of larval PGC cyst proliferation under an irregular food supply condition. Under normal breeding conditions with a constant food supply, the percentage of larval ovaries containing PH3-positive proliferating PGC cysts was consistent throughout the L3 stage (22.0%, *n* = 41, Fig. 4A, F). However, no L3 ovary (0%, *n* = 23) examined contained PH3-positive PGCs after 3-day starvation (Fig. 4B, F), indicating proliferation arrest of PGCs. Few ovaries (2.3%, *n* = 88) contained PH3-positive PGC cysts at 8 h after refeeding (Fig. 4C, F). Notably, the ovaries resumed normal division rates 16 h (21.4%, *n* = 42) and 24 h (20.0%, *n* = 70) after refeeding (Fig. 4D–F), indicating a swift response to the availability of food. Likewise, L2 ovaries behaved similarly in the starvation/refeeding experiments (Additional file 7: Fig. S4A-F). These data suggest that a cyst-like proliferation strategy may provide flexibility to the regulation of PGC proliferation in response to food availability.

In both vertebrates and invertebrates, TOR signaling is the key regulator of nutritional status, cellular growth, and metabolism in response to environmental inputs [36–38]. Hence, we investigated whether the prompt response of PGC cyst to nutrients is mediated by TOR signaling using rapamycin, an inhibitor of TOR protein kinase. Notably, only 9.3% of L3 ovaries with rapamycin treatment (*n* = 75) showed PH3-positive PGC cysts, compared to 26.9% in mocked-treated ovaries (*n* = 52) (*p* = 0.0139; Fig. 4G–I). Similarly, a significant drop of proliferating ovaries from 16.5% in mock-treated (*n* = 85) to 4.8% in rapamycin-treated (*n* = 84) L2 larvae was observed (*p* = 0.0226; Additional file 7: Fig. S4G-I). Together, these results suggest the involvement of TOR signaling in regulating PGC cyst proliferation in response to nutritional status.

Next, we examined the effects of larval nutritional status on adult fecundity. To this end, we developed a long-term starvation regime, with a minimal amount of food supply during larval growth. Indeed, the ovaries in long-term starved larvae were significantly smaller than those in control larvae and contained fewer PGCs (Fig. 4J, K). In line with this, adult ovaries from the larval starvation group contained fewer ovarioles (19.2 ± 0.8, *n* = 16) than those from controls (67.7 ± 1.8, *n* = 11, *p* < 0.0001; Fig. 4L–N). Consistently, a significant reduction of an average egg number produced by the starvation group (25.6 ± 1.2, *n* = 93) was observed, in comparison with the control group (102.6 ± 5.3, *n* = 40, *p* < 0.0001; Fig. 4O). Of note, the hatch rate of eggs from the starvation group was comparable to that of the control group (*p* = 0.3144; Fig. 4P). Collectively, these results show that nutritional status during the larval stage has a strong impact on ovarian development, as well as on the fecundity of adult mosquitoes.

In summary, *Ae. aegypti* evolves in a germline cyst-like proliferation manner during the larval stage to regulate PGC dynamics, presumably via the TOR pathway, in response to nutritional status, which is also important for its fecundity.

### Ovariole is formed via a distinct mode during metamorphosis in* Ae. aegypti*

**Fig. 5:**
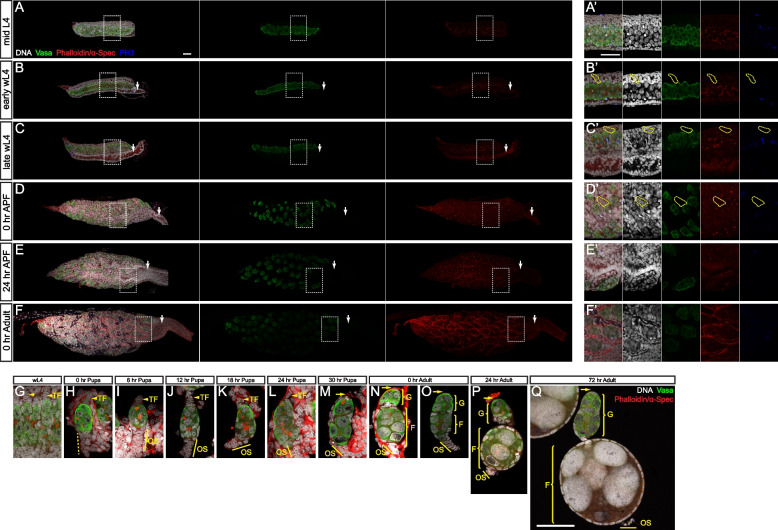
Behavior of TF stacks during ovarian formation. Confocal images (**A**–**F**) and zoomed-in for outlined areas (**A’**–**F’**) of the ovaries of indicated developmental stages. Vasa (green), α-Spec/phalloidin (red), DNA (white), and PH3 (blue) staining. **A**, **A’** PGC mass elongates along the A/P axis during mid-L4. **B**, **B’** Terminal filament formation and branched fusomes breakdown occur during early wL4. **C**, **C’** PGC mass breakdown into 2–6 clusters and pre-ovariole formation during late wL4. **D**, **D’** Pre-ovarioles grow and migrate. **E**, **E’** Pre-ovarioles continue to grow and migrate with ovariole stalks pointing toward the oviduct. **F**, **F’** Pre-ovarioles develop into two segments (indicated by a yellow rectangle). Confocal images of pre-ovarioles/ovarioles of wL4 (**G**), 0 h pupa (**H**), 6 h pupa (**I**), 12 h pupa (**J**), 18 h pupa (**K**), 24 h pupa (**L**), 30 h pupa (**M**), 0 h adult with (**N**) or without (**O**) muscle sheath, 24 h adult (**P**), and 72 h adult (**Q**) with α-Spec/phalloidin (red) and DNA (white) staining show the development of terminal filament (TF), ovariole stalk (OS), germarium (G), and the primary follicle (F). White arrows in **B**–**F** mark the oviducts. Yellow dashed lines in **B’**–**D’** mark one TF. Yellow arrowheads in **G**–**Q** mark the TF position. Yellow dash line in **H** marks the developing OS. Yellow lines in **I**–**Q** mark the OS. Scale bars in **A** (for **A**–**F**), **A’** (for **A’**–**F’**), and **Q** (for **G**–**Q**) indicate 50 µm

In *D. melanogaster*, ovariole formation starts with the formation of TF at the apical side of the ovariole, and the number of TF stacks predetermines the ovariole number [39]. Although the overall structure of *Ae. aegypti* ovariole is similar to that of *D. melanogaster*, to our surprise, no TF stack equivalents were observed in adult ovarioles (Fig. 5N–Q). Hence, we investigated how *Ae. aegypti* ovarioles are formed in detail.

During the mid-L4 stage, a PGC mass was asymmetrically surrounded by somatic cells, with one side covered by a single layer of somatic cells and the other side enclosed with multiple layers (Fig. 5A, A’). During the early wL4 stage, the ovary grew, and PGC mass elongated to form a long stretch along the A/P axis (Fig. 5B, B’). Subsequently, somatic cells of the multiple layers underwent a reorganization to form multiple stacks, the TF stack equivalents (marked by yellow dashed lines), with one end attaching to the PGC stretch (Fig. 5B, B’). During the late wL4 stage, the PGC mass broke down into smaller clusters which contain 2–6 PGCs; meanwhile, extensively branched fusomes were also fragmented into spectrosomes (Fig. 5C, C’). Some somatic cells migrated to enclose those 2–6 PGC clusters to form pre-ovarioles (Fig. 5C, C’). One narrow cavity, the developing oviduct, was evident between the PGC stretch and the single layer of somatic cells (Fig. 5B, C and Additional file 8: Fig. S5A). During the pupal stage, the PGC number increased, and the pre-ovarioles contained more PGCs (Fig. 5D, D’). Notably, those pre-ovarioles also underwent extensive migration and rotation, a process not observed during *D. melanogaster* ovariole formation. During wL4, the pre-ovarioles are formed with TF stacks pointing to the lateral side of the ovary, orthogonal to the A/P axis of the ovary (Fig. 5C, C’). Concomitant with ovarian growth, pre-ovarioles migrated and resided on the surface of the ovary with an oviduct located in the central position of the ovary (Fig. 5D, E). Meanwhile, pre-ovarioles underwent about a 90° rotation from the lateral to the A/P orientation (Fig. 5D, D’, E, E’, F, F’).

Although TF stacks were observed during ovariole formation (Fig. 5C, G), no TF stacks were observed in adult ovarioles (Fig. 5N–Q). During wL4, TF stacks formed on one side of the elongated PGC mass before fusome breakdown and the formation of pre-ovarioles (Fig. 5G). During larval/pupal transition, PGCs of pre-ovarioles increased without differentiation (Fig. 5G–I). Meanwhile, somatic cells on the basal side (opposite end of TF) intercalated to form an ovariole stalk (equivalent to the basal stalks in *D. melanogaster*), which connects pre-ovarioles to developing oviduct (Fig. 5H–I). During the late pupal stage, PGCs continued to increase, and the ovariole elongated to form two segments: the germarium and the developing primary follicle, separated by somatic cells in between (Fig. 5J–M). The germarium contained spectrosome-containing germ cells, while the developing primary follicle harbored an 8-cell cyst (Fig. 5M). Of note, the TF stack underwent a morphological change and became thin during the late pupal stage (Fig. 5K–L) and was eventually degenerated 30 h after pupa formation (Fig. 5M). Consequently, adult ovarioles did not have TF stacks attached to the germaria (Fig. 5N–Q), indicating a complete loss of TF during development. After emergence, the ovarioles underwent a maturation process. First, the primary follicle separated from the germarium, grew, and developed to the resting stage (Fig. 5N–P). Second, a new 8-cell cyst emerged from the germarium (Fig. 5Q). Third, the ovariole stalk appeared to undergo a morphological change and became relatively rounded in shape (Fig. 5O–P).

Collectively, *Ae. aegypti* exhibited distinct features during ovariole formation, including PGC mass elongation and breakdown, pre-ovariole migration and rotation, and TF stack degeneration during metamorphosis, in comparison with *D. melanogaster*.

### Ovariole formation is triggered by ecdysone signaling

**Fig. 6:**
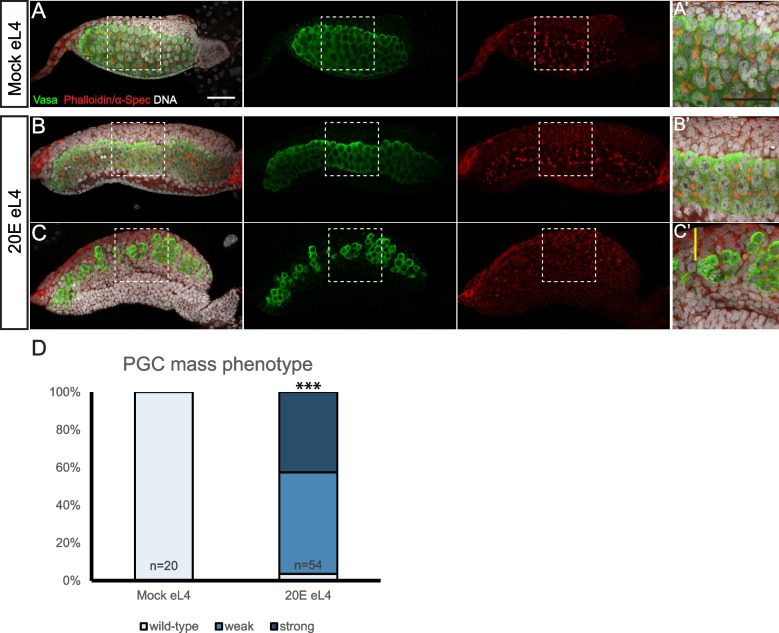
Ecdysone signaling triggers the ovariole formation during metamorphosis. Confocal images (**A**–**C**) and zoomed for outlined areas of eL4 ovaries with mocked treatment (**A**, **A’**) and 20E treatment (**B**, **B’**, **C**, **C’**) with Vasa (green), α-Spec/phalloidin (red), and DNA (white) staining. **A**, **A’** Mock-treated ovary shows wild-type structure. **B**, **B’**, **C**, **C’** 20E-treated ovary exhibits precocious developmental features, including PGC mass elongation (weak phenotype, **B**, **B’**) and pre-ovariole formation (strong phenotype, **C**, **C’**). **D** Quantification of percentages of the ovaries with wild-type, weak, and strong phenotypes from three pooled independent replicates using the chi-squared test. The number of samples (*n*) in each group is shown above the *X*-axis. ***p < 0.001. Yellow line in **C’** marks one TF stack. Scale bar indicates 50 µm

In *Ae. aegypti*, the ovariole formation begins from the wL4 stage and continues throughout the pupal stage, concurrent with metamorphosis, a critical developmental transition from aquatic larvae to terrestrial adults. It has been well known that the steroid hormone ecdysone is the master regulator of this transition during insect development [40, 41]. In addition, ecdysone signaling involves in the establishment of stem cell niche during the ovariole formation in *D. melanogaster* [42]. We thus investigated whether the formations of TF stacks and pre-ovarioles are triggered by the ecdysone hormone. To induce an early onset of ecdysone pulse mimicking larvae/pupae transition, we subjected larvae at different developmental stages to a pulse treatment of biologically active ecdysteroid 20-hydroxyecdysone (20E). Similar to those untreated ovaries, mocked-treated eL4 ovaries contained an intact oval PGC mass (100.0%, *n* = 20; Fig. 6A, A’, D). However, 20E-treated eL4 ovaries exhibited precocious PGC mass elongation (weak phenotype, 53.7%, *n* = 54; Fig. 6B, B’, D), TF formation and PGC mass breakdown (strong phenotype, 42.6%, *n* = 54; Fig. 6C, C’, D), and hallmarks of precocious pre-ovariole formation, which is significantly different from the mock control (*p* < 0.0001). In contrast, when L2 or L3 larvae were subjected to the same treatment, no noticeable change in ovarian development was observed (Additional file 9: Fig. S6A-D), suggesting inhibitory mechanisms in place to prevent 20E-mediated PGC mass breakdown during early developmental stages or prerequisites of additional developmental processes for 20E function during L4 stage. These data showed that the early onset of ecdysone pulse during eL4 is sufficient to induce a precocious PGC mass breakdown, suggesting that ovariole formation is triggered by ecdysone signaling in *Ae. aegypti*.

### Ovarian development is conserved in mosquitoes

**Fig. 7:**
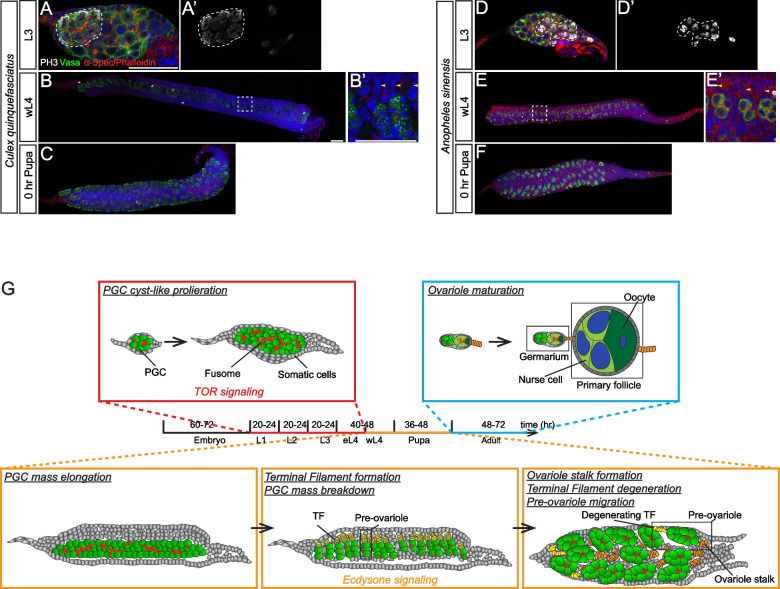
Ovarian development in *Culex* and *Anopheles* and illustration of ovary development in mosquitoes. Confocal images of L3 (**A**, **A’**), wL4 (**B**, **B’**), or 0 h pupa (**C**, **C’**) ovary of *Culex quinquefasciatus*. Confocal microscopy images of L3 (**D**, **D’**), wL4 (**E**, **E’**), or 0 h pupa (**F**) ovary of *Anopheles sinensis*. Vasa (green), α-Spec/phalloidin (red), DNA (blue), and PH3 (white) staining. **A**, **A’**, **D**, **D’** PGC mass contains PH3-positive PGC cyst. **B**, **B’**, **E**, **E’** wL4 ovary shows terminal filament formation, fusome breakdown, and PGC mass breakdown. **C**, **F** Pre-ovarioles undergo proliferation and migration during the pupal stage. The dash lines in **A**, **A’**, **D**, and **D’** mark the PH3 + PGC cysts. Yellow arrows mark the TF stacks. Scale bars in **A** (for **A**, **A’**, **D**, **D’**), **B** (for **B**, **C**, **E**, **F**), and **B’** (for **B’**, **C’**) indicate 50 µm. **G** A scheme shows the processes of ovary development in mosquitoes, including unique PGC cyst-like proliferation and ovariole formation

So far, our data revealed a novel mode of insect ovarian development in *Ae. aegypti*, which exhibits two main unique features, a PGC cyst-like proliferation during larval development and a distinct process of ovariole formation during metamorphosis (Fig. 7G). To test if this strategy is conserved in other mosquito species, we examined the ovarian development in *Culex quinquefasciatus* and *Anopheles sinensis*. Of interest, in both species, the ovary contained a “PGC mass” and underwent cyst-like proliferation with a mitotic synchrony during the larval stage (Fig. 7A, D). Furthermore, the PGC mass elongation, TF formation, and PGC mass breakdown were observed during ovariole formation (Fig. 7B, E). Lastly, pre-ovarioles also underwent a massive migration and rotation to form an ovarian structure during the pupal stage (Fig. 7C, F). Hence, this mode of ovarian development appears to be conserved in various mosquito species.

## Discussion

In this study, we report the ovarian development of *Ae. aegypti* (Fig. 7G) by comparing it with that of *D. melanogaster*, a well-studied Dipteran model. Our study shows some conserved developmental features but also identifies some unexpected and distinct events during *Ae. aegypti* ovarian development, revealing an alternative mode of ovarian development in insects.

### Conserved germline development during the embryonic stage

The establishment of PGCs is the initiative event of germline development. Two modes of PGC formation, maternal provision and zygotic induction, have been documented in insects [43, 44]. In *D. melanogaster*, PGCs are specified by maternal deposits, Vasa-containing “germ plasm,” at the posterior end of embryos. Similarly, we observed a posteriorly localized Vasa crescent in early embryos, supporting the “maternal provision” mode of germ cell specification in *Ae. aegypti* [4]. PGCs located at the extra-embryonic space undergo a conserved migration route to form gonads during embryonic development in *Ae. aegypti*, resembling the processes observed in *D. melanogaster*. In *D. melanogaster*, about 30 PGCs form, and only about 12 PGCs arrive at each embryonic gonad [27, 45], while in *Ae aegypti*, Raminani and Cupp observed about 60% of pole cells successfully migrate to form embryonic gonads, each hosting 4–6 PGCs [14, 15]. Indeed, we detected those mismigrated PGCs by Vasa staining during the PGC migration, a similar event was reported during *D. melanogaster* PGC migration [25, 26]. These migrating PGCs are mitotic inactive but resume to proliferate after gonad formation in both *Ae. aegypti* and *D. melanogaster* [6]. In summary, our data support the conserved features of germline development, including PGC formation, migration, and proliferation during the embryonic stage between *Ae. aegypti* and *D. melanogaster* in Diptera [44].

### Different modes of PGC proliferation

PGCs proliferate to generate sufficient cell numbers before ovariole formation. *D*. *melanogaster* PGCs are surrounded and separated from each other by somatic ICs, which regulate PGC proliferation [27]. These PGCs are independent entities with a distinct proliferation program and undergo asynchronized divisions. Of surprise, *Ae. aegypti* utilizes a germline cyst-like proliferation manner to expand its PGC pool. Through incomplete cytokinesis, PGCs form a tightly packed PGC mass consisting of several PGC cysts without somatic ICs. PGCs in each cyst share cytoplasm and divide synchronously. Such cyst-like proliferation mode during early PGC expansion has not been reported in insects but, to the best of our knowledge, only documented during embryonic testis development in mice [34].

The connected cyst division pattern, however, is well observed during gametogenesis in adult metazoans [46]. During the oogenesis of the meroistic type of insect ovary, nurse cells support oocyte development within the germ cell cyst. While during animal spermatogenesis, X-chromosome- and Y-chromosome-containing spermatids support each other’s development through a shared cytoplasm. However, the biological significance of this unique cyst-like proliferation mode during PGC proliferation remains elusive. One possible explanation is that a fine-tune of PGC dynamics could be achieved more promptly in a coordinated way, compared to individually dividing PGCs. Several lines of evidence indeed support this hypothesis. First, larval PGC proliferation exhibits a rapid response to environmental cues (food availability). Larval PGCs cease proliferation upon short-term starvation, yet they can resume a similar proliferating rate as control within a short period of time after refeeding. Second, under prolonged starvation conditions, some PGC cysts undergo apoptosis while others still survive, indicating effective communications might exist among these PGC cysts to coordinate their development. Third, rapamycin treatment experiments suggest that the TOR pathway might be the main signaling to regulate PGC proliferation in response to nutritional status. Collectively, *Ae. aegypti* PGCs adopt this cyst-like division mode to regulate PGC proliferation swiftly and coordinately, in response to intrinsic regulation and environmental challenges. Importantly, a similar germline cyst-like PGC proliferation pattern is also observed in both *Culex* and *Anopheles* mosquitoes, suggesting that it may represent a new PGC proliferation mode in insects.

### Regulation of ovariole reproductive activity

During *D. melanogaster* germline development, ovariole formation is initiated by the establishment of TF stacks from the apical somatic cells of the ovary which is commonly observed in many other insects [1]. In addition to attaching the ovary to the fat body, these TF stacks also direct the migration of apical somatic cells to separate PGCs to form individual ovarioles [8, 47]. Consistent with this, *D. melanogaster bric á brac* mutant, which disrupts TF stack formation, lacks ovarioles [47]. In *Ae. aegypti*, although transient TF stacks are observed during the formation of pre-ovarioles, these TF stacks surprisingly degenerate and are obliterated during pupal development, resulting in no TF stack in adult ovarioles.

The lack of TF stacks in adult ovarioles might potentially explain the different reproductive behavior of ovarioles between *Ae. aegypti* and *D. melanogaster*. *D. melanogaster* female continuously produces eggs throughout most of its lifespan which is supported by self-renewing germline stem cells (GSCs) in the germarium. These GSCs reside in a niche composed of three types of somatic cells: TF, cap cells (CpCs), and escort cells (ECs). Among these cells, CpCs are the main signal source to promote GSC self-renewal [48]. Of interest, CpCs are recruited and specified by TF, suggesting a relay mode of niche establishment. These cell types can be distinguished by their distinct nuclear morphologies (TF cells have disc-shaped nuclei, CpCs contain small and round nuclei, and ECs harbor triangular nuclei). While *D. melanogaster* ovariole normally contains 6–7 developing follicles at various developmental stages, *Ae. aegypti* ovariole harbors only 1–2 follicles. Such ovariole structure (a reduction in follicle number) is widely observed in the lower dipterans [1]. Furthermore, *Ae. aegypti* follicles are developmentally arrested before a blood meal. Each *Ae. aegypti* ovariole produces only one egg after a successful blood meal. In general, *Ae. aegypti* females produce several (~ 3–5) batches of progenies throughout their lifespan, suggesting a low ovariole reproductive activity, the reported feature for the lower dipterans. These further suggest that the low reproductive activity might not be supported by GSCs (as there are a sufficient number of germ cells in the germarium to support the development of 3–5 follicles) or GSC activity is differentially regulated from that of *D. melanogaster*. In addition to the lack of TF stacks, no small and round nucleus cells were observed in *Ae. aegypti* adult ovarioles, and only one type of somatic cells with elongated nuclei was detected at the anterior half of the germarium in which oogonial cells reside (Fig. 5Q). Interestingly, the ovariole of the earwig *Opisthocosmia silvestris*, belonging to the order of Dermaptera, also does not contain CpCs [49]. Hence, it would be interesting to investigate whether *Ae. aegypti* ovariole reproductive activity is supported by resident GSCs and, if so, how GSC activity is regulated. Since it has been well-established that several signaling pathways, including the insulin, ecdysone, and juvenile hormone pathways, play important roles in egg development and maturation, it would be interesting to address whether these signaling pathways also play a role in regulating the activity of these oogonia (or putative GSCs) [10–13].

### An alternative mode of insect ovariole development

Diptera is traditionally classified into two major groups: the mosquito-like lower Diptera “Nematocera” and the higher Diptera “Brachycera” [50, 51]. The thorough knowledge of insect ovarian development mainly comes from studies on *D. melanogaster*, a higher Dipteran in Brachycera suborder. Similar to other dipterans, the *D. melanogaster* ovary consists of polytrophic meroistic type ovarioles with each follicle containing both nurse cells and an oocyte [3]. Although the overall steps for ovarian development (including pole cell formation, PGC migration, ovariole formation, and adult oogenesis) of other dipterans follow similar processes identified in *D. melanogaster* [1], some unique features have been reported in the lower dipterans such as *Tinearia alternata* Say in Psychodidae [51, 52]. In nematoceran, some species contain short TFs and relatively short ovarioles with synchronous development. In addition, some unique features of ovarian development have been reported, such as germ cell divisions, a cluster formation, and a massive cell migration prior to ovariole formation [52–54]. In this study, our results show that the germline development of *Ae. aegypti*, an emerging model for studying vector and pathogen interaction, exhibits both similarity (PGC formation and migration during the embryonic stage) and divergence (PGC proliferation mode, PGC mass breakdown, TF cell degeneration, and ovariole migration) to that of *D. melanogaster*. Of note, *Ae. aegypti* ovarioles do not contain TFs and are structurally different from those of *D. melanogaster*. This type of ovarioles (lacking TF stacks) might reflect their reproductive behavior (see above). Of note, these unique developmental processes are also observed during ovarian development in *Culex* and *Anopheles* mosquitoes, and their adult ovarioles do not have TF stacks either. Thus, the ovarian development of *Ae. aegypti* described here might represent an alternative mode of ovarian development in the lower Diptera, and it would be interesting to investigate if these newly identified developmental features are conserved among insects of the lower Diptera.

## Conclusions

*Ae. aegypti*, a major vector of various disease-causing pathogens that inhabits tropical and subtropical regions, represents a model of lower Diptera. Compared with *D. melanogaster*, a well-studied model of higher Diptera, *Ae. aegypti* utilizes a distinct mode to form functional ovaries during the late stage of larval/pupal stages, including germ cell cyst-like PGC proliferation and ovariole formation. Our study hence reveals an alternative mode of ovarian development in mosquitoes, providing insights into a better understanding of the reproductive system and evolutionary relationship among insects.

## Methods (Table 1)

**Table 1:** Resource used in this study

Reagent or resource	Source	Identifier
**Antibodies**		
Guinea pig polyclonal anti-AaegVasa	This paper	N/A
Rabbit polyclonal anti-cleaved Caspase3 (D175)	Cell Signaling Technology	Catalog # 9661: RRID: AB_2341188
Mouse monoclonal anti-histone H3 (phospho S10)	Abcam	Catalog # ab14955; RRID: AB_443110
Rabbit polyclonal anti-Dm-α-Spec	Liu et al., 2010[55]	N/A
Alexa Fluor 488 AffiniPure goat anti-mouse IgG (H + L)	Jackson ImmunoResearch Laboratories	Catalog # 115–545-146; RRID: AB_2307324
Goat anti-guinea pig IgG (H + L) highly cross-adsorbed secondary antibody, Alexa Fluor 488	Invitrogen	Catalog # A-11073; RRID: AB_2534117
Cy3 AffiniPure goat anti-rabbit IgG (H + L)	Jackson ImmunoResearch LaboraInss	Catalog # 111–165-003; RRID: AB_2338000
Goat anti-mouse IgG (H + L) cross-adsorbed secondary antibody, Alexa Fluor 405	Invitrogen	Catalog # A-31553; RRID: AB_221604
DyLight 405 AffiniPure goat anti-rabbit IgG (H + L)	Jackson ImmunoResearch Laboratories	Catalog # 111–475-003; RRID: AB_2338035
**Chemicals**		
TO-PRO-3 iodide (642/661)	Invitrogen	Catalog # T3605
Hoechst 33,342	Invitrogen	Catalog # H21492
Alexa Fluor 546 phalloidin	Invitrogen	Catalog # A22283
Click-iT EdU Cell Proliferation Kit for Imaging, Alexa Fluor™ 555 dye	Invitrogen	Catalog # C10338
20-Hydroxyecdysone	Sigma-Aldrich	Catalog # H5142
Rapamycin	LC Laboratories	Catalog # R-5000
**Experimental models: organisms/strains**		
* Aedes aegypti* (NEA-EHI strain)	EHI, NEA	N/A
* Aedes aegypti* (Liverpool strain)	Chun Hong Chen Lab, NHRI	N/A
* Culex quinquefasciatus* (NEA-EHI strain)	EHI/NEA	N/A
* Anopheles sinensis* (NEA-EHI strain)	EHI, NEA	N/A
**Software and algorithms**		
GraphPad Prim 6	GraphPad Software	RRID:SCR_002798
FIJI	Open source [56]	RRID:SCR_002285
Illustrator	Adobe	RRID:SCR_010279
Photoshop	Adobe	RRID:SCR_014199
Leica Application Suite X	Leica	RRID:SCR_013673
**Oligonucleotides**		
AaegVasa_EcoR1 F	GCGGATCCTTCGGCGGTGGCGACAATGA	
AaegVasa_Xho1 R	CGCTCGAGTCAGGTCTGGGCACAGGCCATTA	
Aaegα-Spec T7 F1	TAATACGACTCACTATAGGGATGGAACAGTTTACCCCCAAGGAGG	
Aaegα-Spec T7 R1	TAATACGACTCACTATAGGGCTGTTGCAATTTCATTCCCTTTTCG	
Aaegα-Spec T7 F2	TAATACGACTCACTATAGGGGAACACCGTACCGAGGTAGATGCCCGC	
Aaegα-Spec T7 R2	TAATACGACTCACTATAGGGACGATCCAACACATCTGCACGCTTGTCG	

### Mosquito breeding

*Ae. aegypti* (NEA-EHI strain and Liverpool strain), *Culex quinquefasciatus* (NEA-EHI strain), and *Anopheles sinensis* (NEA-EHI strain) were reared in the environmental chamber (28 °C, 80% relative humidity, 12L: 12D photoperiodic regime). *Ae. aegypti* eggs (2–4 weeks old) were hatched in sterile water using a vacuum for 15 min. Two hundred newly hatched L1 larvae were transferred into a plastic container with 500 mL of sterile water. Mosquito larvae were fed a mixture of fish food/brewer’s yeast (2:1 w/w) daily, with 50 mg (day 1), 100 mg (day 2), 200 mg (day 3), 300 mg (day4), 300 mg (day 5), and 200 mg (day 6). *Culex quinquefasciatus* and *Anopheles sinensis* eggs hatched naturally in the water. Two hundred newly hatched L1 larvae were transferred into a plastic container with 500 mL of sterile water and fed with fish food daily. Male/female pupae were collected separately. An equal number of newly emerged males and females were transferred into the cage together supplied with one bottler of 10% sugar solution and one bottle of water. Mature misquotes (3–5 days after emergence) were fed with rabbit blood using an artificial membrane feeding system (Hemotek Ltd., UK) to produce eggs.

### Developmental staging

Under our breeding condition, *Ae. aegypti* (Liverpool and NEA-EHI strains) took 60–72 h for embryogenesis; 20–24 h for L1, L2, and L3; 40–48 h for L4; and 40–48 h for pupae before their emergency as an adult (Fig. 1A).

For L1–L3 stages, we chose middle-aged larvae to examine the germline development (10 h after larval hatching, ALH for L1, 30 h ALH for L2, and 50 h ALH for L3). For L4, we divided it into two phases, 60–80 h ALH as early L4 (eL4) and 80–100 h ALH as wandering L4 (wL4), and checked their germline at 70 h and 90 h ALH, respectively.

### Starvation/refeeding assay

Larvae of desired age were rinsed with sterile water and kept in fresh sterile water that changed daily. Before refeeding with fish food/brewer’s yeast (2:1 w/w), L2 or L3 larvae were starved for 2 days or 3 days, respectively. L2 or L3 were dissected at 0 h/8 h/16 h/24 h after refeeding.

### Rapamycin treatment

Larvae of desired age were transferred into a 6-wll plate containing 0.1% DMSO (solvent control) or 200 µM rapamycin (stock is dissolved in DMSO) in sterile water with sufficient food for 16 h. Treated larvae were subsequently subject to dissection and immunostaining procedures.

### 20E treatment

Larvae of desired age were rinsed with sterile water and transferred into a 24-well plate containing 1% EtOH (solvent control) or 1 mM 20E (stock is dissolved in EtOH) in sterile water with sufficient food for 16 h. Treated larvae were subsequently rinsed with sterile water 3 times and transferred back to a container with sterile water and food for further development. Four hours later, treated larvae were subject to dissection and immunostaining procedures.

### Edu treatment

Larvae of desired age were rinsed with sterile water and transferred into a 1.5-mL Eppendorf tube containing 1 mM Edu for 2 h. Treated larvae were then rinsed with sterile water 3 times and transferred back to a container with sterile water and food for further development. After the immunostaining steps, the Edu labeling reaction was conducted according to the manufacturer’s protocol (Click-iT® EdU, Invitrogen).

### Dissection and immunostaining

Mosquito embryos were collected in 15 min period from female adult mosquitoes at 72 h after blood feeding and aged in wet containers. Aged embryos were treated with a dechorionation solution (1 volume 5.25% sodium hypochlorite: 3 volumes of distilled water) for 30 s and washed with sterile water. Treated embryos were fixed with fixation solution (4% Formaldehyde in 0.1% Tween20 in PBS, PBST) and heptane (1:1) for 30 min. After washing, the embryos were incubated with boiled water for 30 s. Embryos were stored in menthol at − 20 °C. The endochorion was peeled off by a sharp needle. The peeled embryos were washed with PBST. Mosquito larvae or adults were dissected in cold PBS and fixed in 4% formaldehyde for 10 min. Samples were immunostained with guinea pig anti-AaegVasa (1:5 k, this study), rabbit anti-cleaved Caspase3 (1:200, D175, Cell Signaling Technology), mouse anti-PH3 (1:10 k, Abcam), and rabbit anti-Dm-α-Spec (1:3 k, [55]). F-actin was labeled by Alexa Fluor™ 555 Phalloidin (1:200, Invitrogen). DNA was labeled by Hoechst or TP-PRO-3. Mounting was performed in VectaShield mounting medium.

### Quantification of egg number and hatching rate

After emergence, 30 wild-type virgin male mosquitos were crossed with 30 virgin females for each group. The mosquito adults were supplied with 10% w/v sucrose solution and one vial of sterile water. Three days later, the mosquitoes were fed with rabbit blood for 3 h. Two days after blood feeding, eggs were collected by one piece of filter and counted. Three days after egg laying, eggs were hatched by vacuuming for 15 min. The hatching rate was calculated by the larva/egg number ratio.

### Generation of an anti-AaegVasa antibody

The sequence corresponding to amino acids 63 to 261 of AaegVasa polypeptide (FGGGDNDGEYQNGYSRGGGGGYGGDDDANGHENGFGGGDRGGFRGRGRGGRGGRGGRGGGRSDFGGGDNEGENGFGRGGGGGGFRSRNDDENNENGTDDQVKTEKPRELYIPPAPTENEDEMFGSGISSGINFDKFDEIKVNVTGENPPSPIKSFGDSGLRDYLLQNIRKSHYTKPTPIQKYAIPIIMDKRDLMACAQT) was amplified by AaegVasa_EcoR1 F and AaegVasa_Xho1 R and cloned into pGEX-4 T-1 vector. GST-fusion protein expressed in *E. coli* strain BL21 and purified before injection to mice and guinea pigs to generate antibodies.

### dsRNA injection

*Ae.aegypti* α-Spectrin homolog AAEL015065 was knockdown via dsRNA embryonic injection. Two sets of primers Aaegα-Spec T7 F1/Aaegα-Spec T7 R1 and Aaegα-Spec T7 F2/Aaegα-Spec T7 R2 were used to amplify the respective target DNA sequences of 450 bp in length with T7 promoter (underlined sequences) from genomic DNA. dsRNAs were synthesized by in vitro transcription using MEGAscript™ T7 Transcription Kit (Cat# AMB1334-5; Invitrogen™) according to the manufacturer’s instructions. Two to 3 µg/µL of dsRNA was used for embryo injection.

### Confocal imaging

Confocal imaging was performed with the SP8 system (Leica) equipped with HCX PL APO oil objective lens (40 × /1.3; Nikon) or HC PL APO air lens (20 × /0.75: Nikon). Images were processed using Leica LASX, Adobe Photoshop, Imaris, and ImageJ.

### Statistical analyses

All statistics were performed using GraphPad Prism version 6 (GraphPad Software). Statistical significance was determined using Fisher’s exact test (Fig. 4F, I, Additional file 7: S4F and S4I), non-parametric *t*-test (Fig. 4N–P), and chi-squared test (Fig. 6D). Error bars in all charts represent SEM. Significance was defined as ***p < 0.001, **p < 0.01, *p < 0.05, and ns as not significant in all graphs.

## Supplementary Information

**Additional file 1:Fig. S1.** PGC formation, gonad formation and PGC migration during embryonic stage, related to Fig. 1.

**Additional file 2:Table S1.** PGC numbers of different larval stage.

**Additional file 3:Fig. S2.** Distinct morphology of ovary and testis during L2 stage, related to Fig. 2.

**Additional file 4:Fig. S3.** Phalloidin, α-Spec, Edu and Cleaved-Caspase3 staining in larval ovaries, related to Fig. 3.

**Additional file 5:Video S1.** 3D reconstruction of L1 gonad.

**Additional file 6:Video S2.** 3D reconstruction of L2 ovary.

**Additional file 7:Fig. S4.** PGC cyst-like division responds to nutrition status promptly during L2 stage, related to Fig. 4.

**Additional file 8:Fig. S5.** Cross sections of late wL4 ovary, related to Fig. 5.

**Additional file 9:Fig. S6.** 20E does not trigger morphological change in L2 or L3 ovaries, related to Fig. 6.

**Additional file 10.** Legends for Additional files 1–9.

## Data Availability

All data generated or analyzed during this study are included in this published article and its supplementary information files.

## Abbreviations

20E: 20-Hydroxyecdysone
*Ae.**aegypti*: *Aedes* *aegypti*
AEL: After egg laying
A/P: Anterior/posterior
CpC: Cap cell
IC: Intermingled cell
*D.**melanogaster*: *Drosophila melanogaster*
EC: Escort cell
eL4: Early L4
ER: Endoplasmic reticulum
GSC: Germline stem cell
IIT: Incompatible insect technique
PGC: Primordial germ cell
PH3: Phosphor-Histone H3
SIT: Sterile insect technique
TF: Terminal filament
TOR: Target of rapamycin
wL4: Wandering L4
