# Supplementary figures and images for: Aedes aegypti exhibits a distinctive mode of late ovarian development
Source: BMC Biol. 2023 Jan 24;21:11. doi: 10.1186/s12915-023-01511-7 (PMC9872435; doi:10.1186/s12915-023-01511-7)

Figure S1

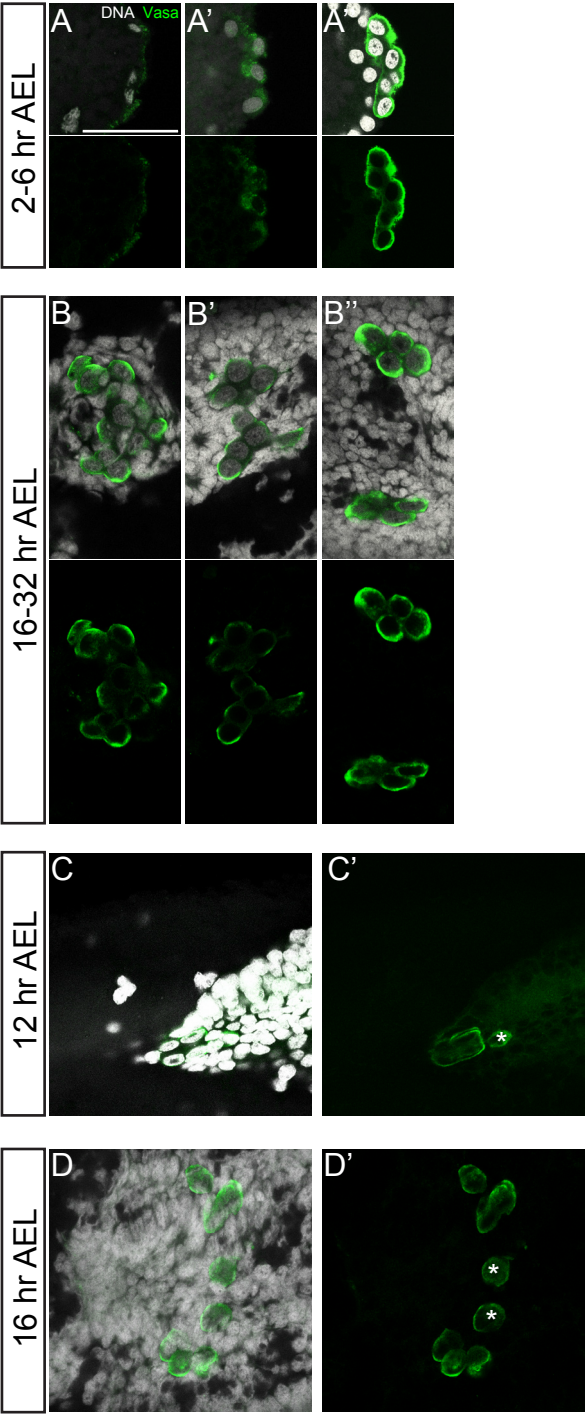

Supplement: Supplementary file 1 — Additional file 1:Fig. S1. PGC formation, gonad formation and PGC migration during embryonic stage, related to Fig. 1. [file 12915_2023_1511_MOESM1_ESM.pdf]

Figure S2

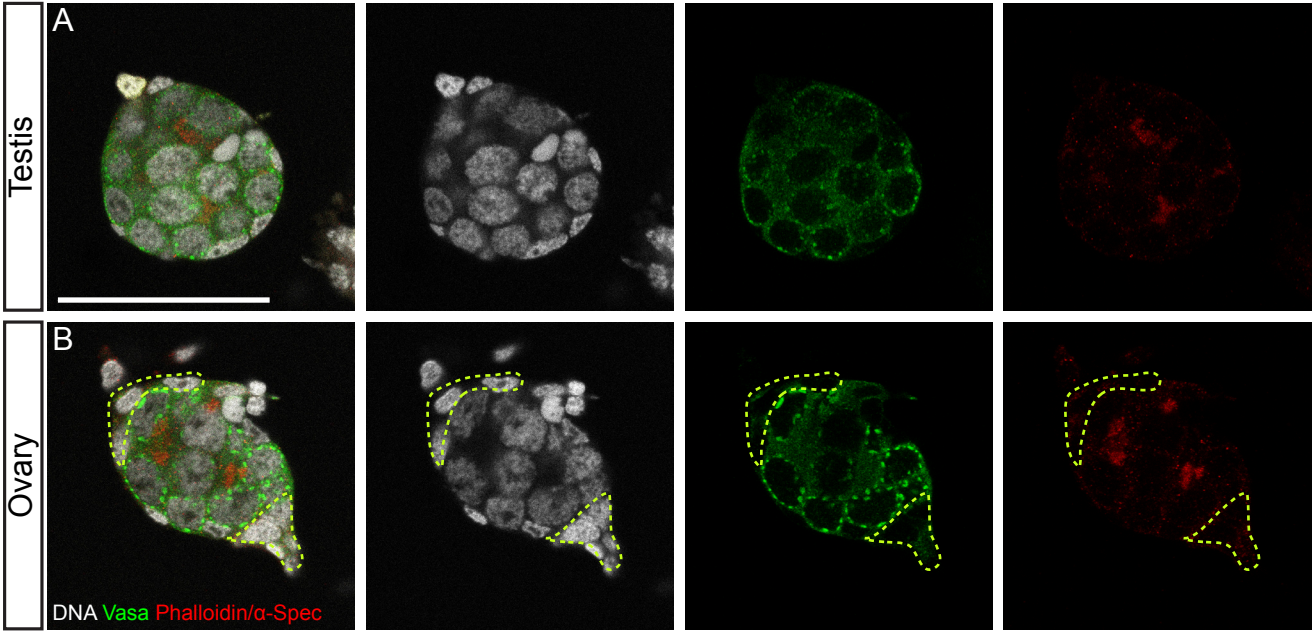

Supplement: Supplementary file 3 — Additional file 3:Fig. S2. Distinct morphology of ovary and testis during L2 stage, related to Fig. 2. [file 12915_2023_1511_MOESM3_ESM.pdf]

Figure S3

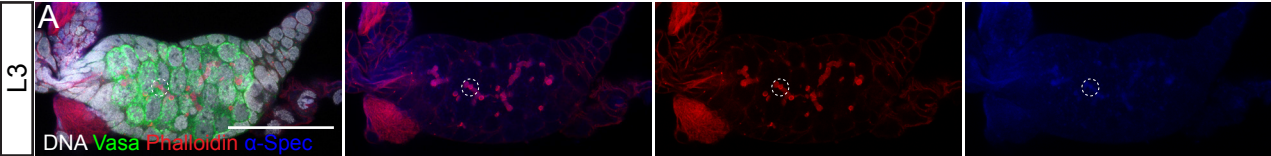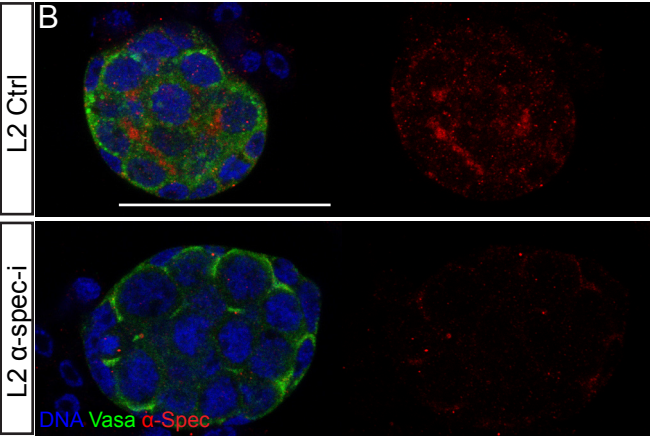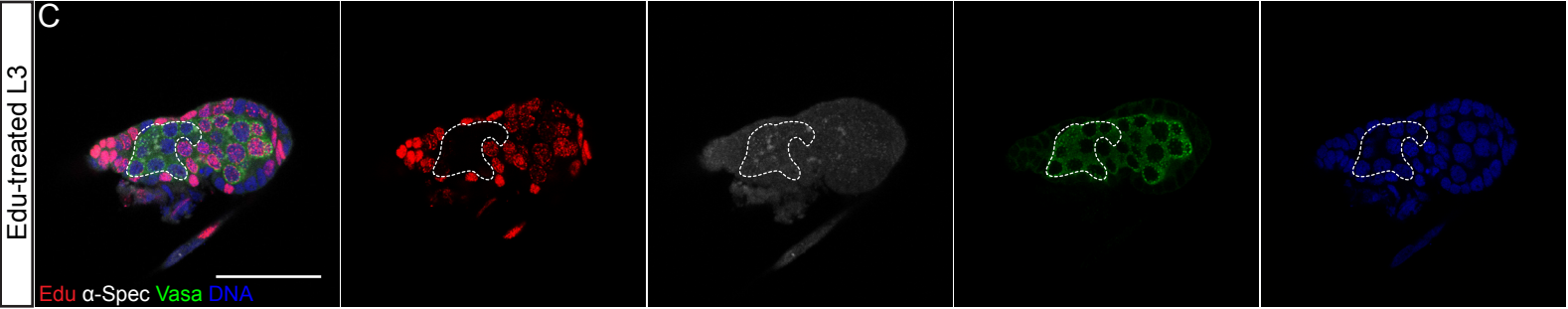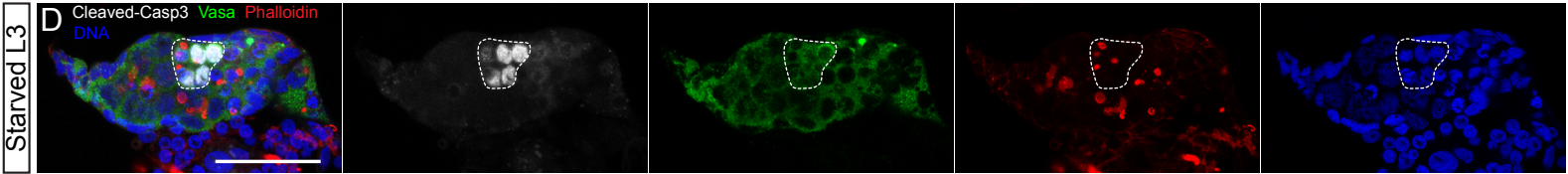

Supplement: Supplementary file 4 — Additional file 4:Fig. S3. Phalloidin, α-Spec, Edu and Cleaved-Caspase3 staining in larval ovaries, related to Fig. 3. [file 12915_2023_1511_MOESM4_ESM.pdf]

Figure S4

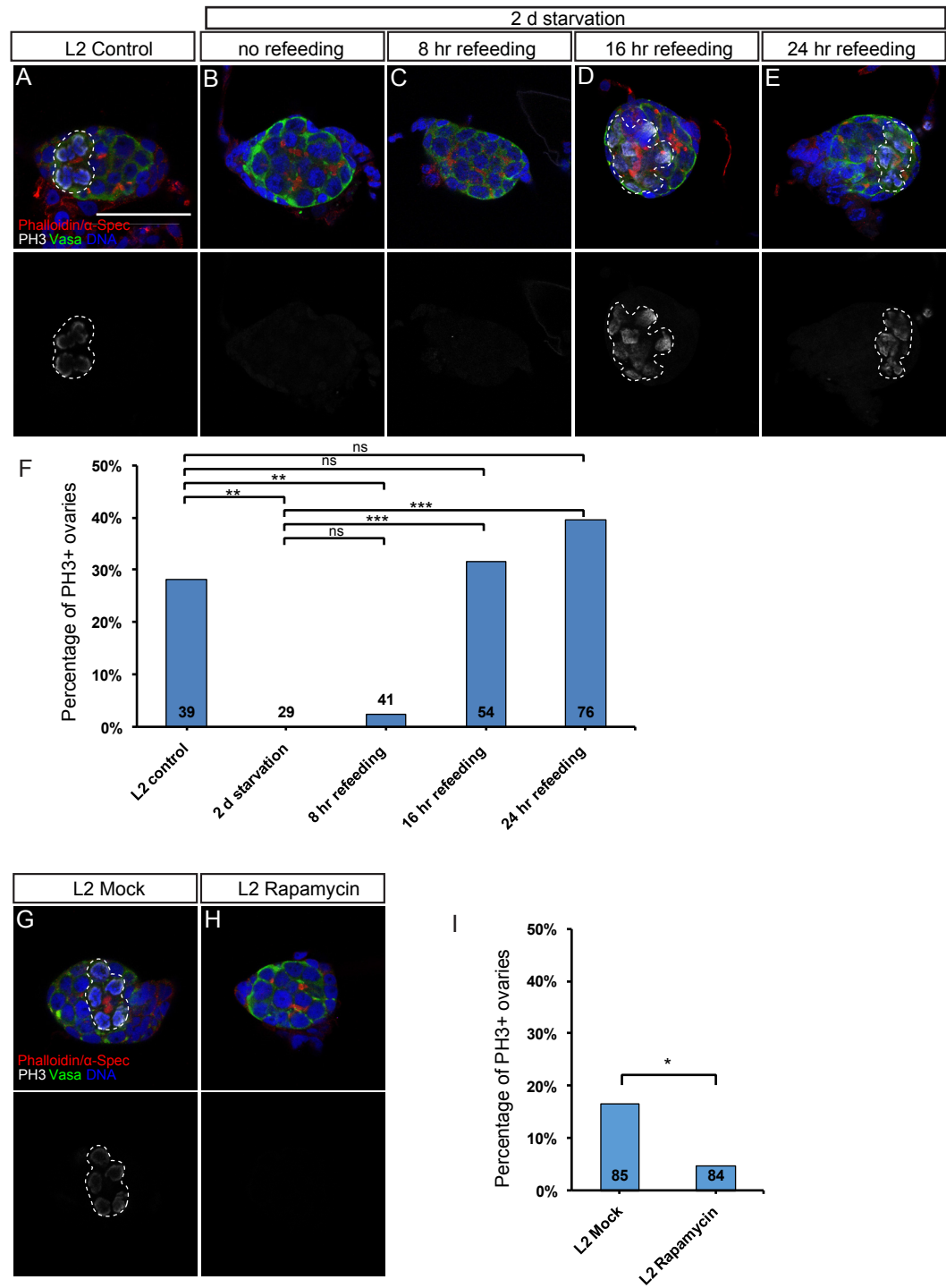

Supplement: Supplementary file 7 — Additional file 7:Fig. S4. PGC cyst-like division responds to nutrition status promptly during L2 stage, related to Fig. 4. [file 12915_2023_1511_MOESM7_ESM.pdf]

Figure S5

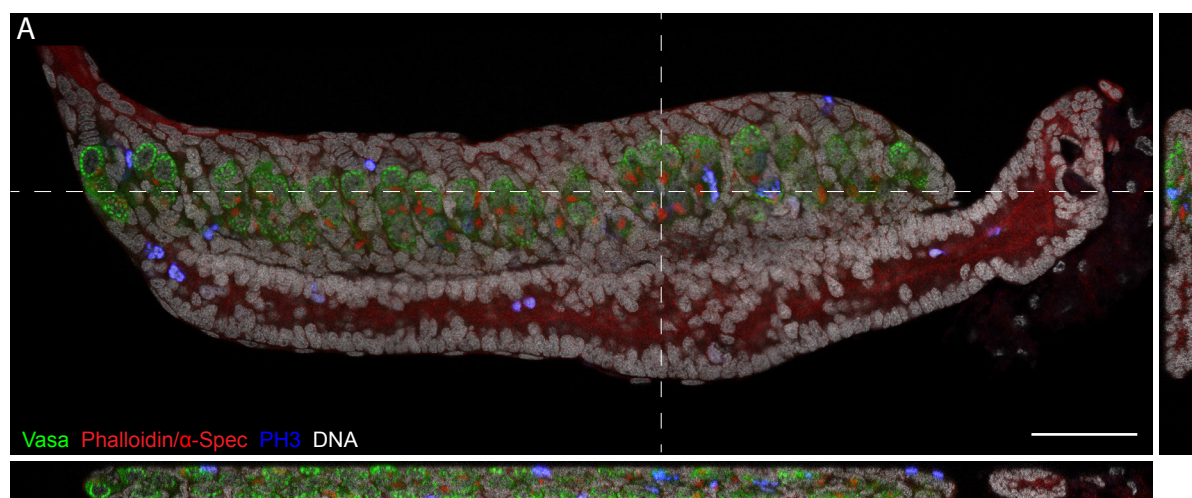

Supplement: Supplementary file 8 — Additional file 8:Fig. S5. Cross sections of late wL4 ovary, related to Fig. 5. [file 12915_2023_1511_MOESM8_ESM.pdf]

Figure S6

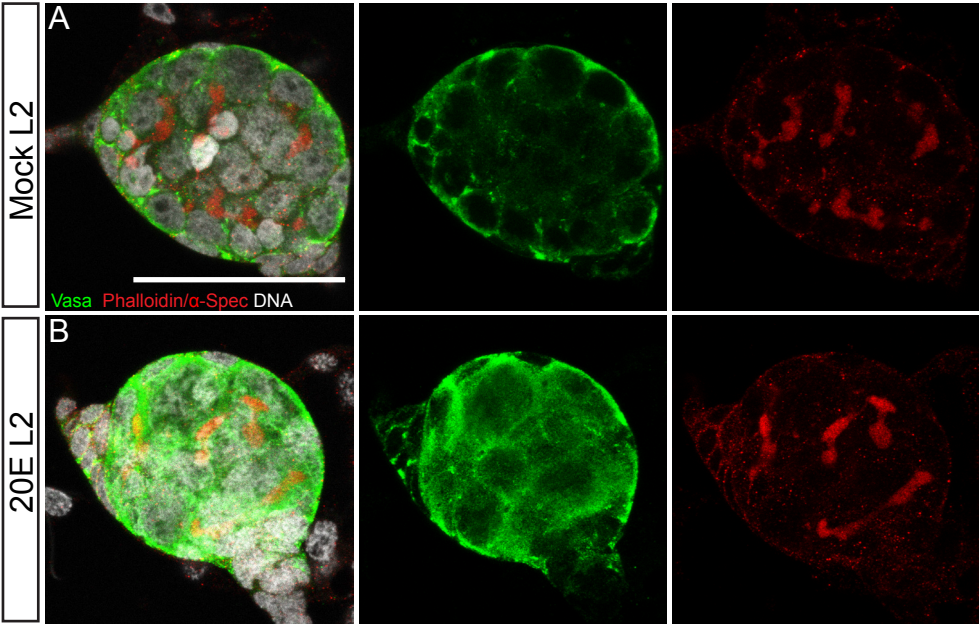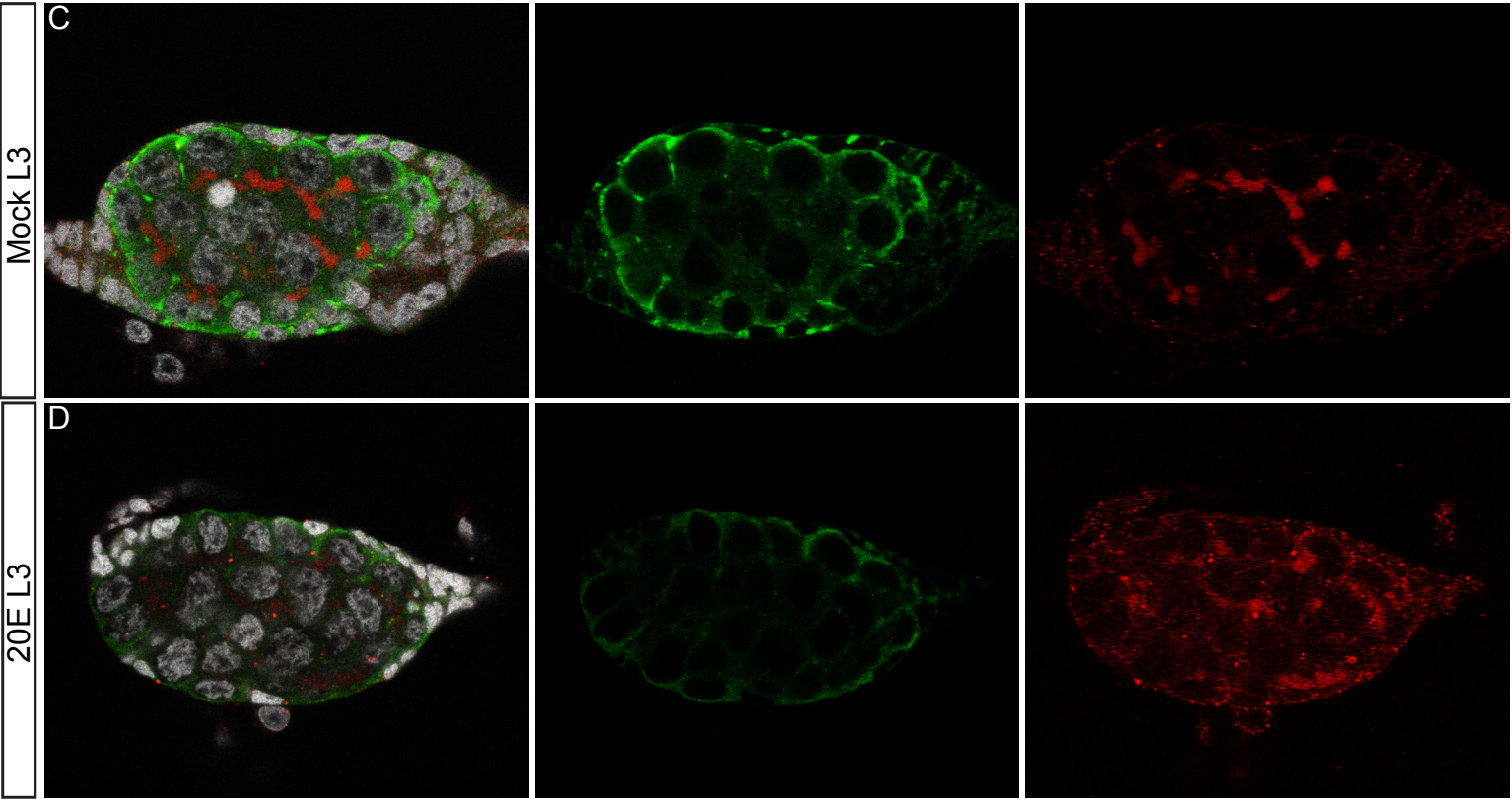

Supplement: Supplementary file 9 — Additional file 9:Fig. S6. 20E does not trigger morphological change in L2 or L3 ovaries, related to Fig. 6. [file 12915_2023_1511_MOESM9_ESM.pdf]
