# Supplementary material for: Aedes aegypti exhibits a distinctive mode of late ovarian development
Source: BMC Biol. 2023 Jan 24;21:11. doi: 10.1186/s12915-023-01511-7 (PMC9872435; doi:10.1186/s12915-023-01511-7)
Supplement: Supplementary file 10 — Additional file 10. Legends for Additional files 1–9. [file 12915_2023_1511_MOESM10_ESM.docx]

**Additional files**

**Additional file 1: Figure S1. PGC formation, gonad formation and PGC migration during embryonic stage, related to Figure 1.**

(A-A’’) Confocal images of embryos from 2 to 6 hr AEL showing the process of PGC formation with Vasa (Green) and DNA (White) staining. First, Vasa is detected at the posterior end of the embryo (A). Second, nuclei form within the Vasa crescent (A’). Third, PGCs form at the posterior end of the embryo (A’’). (B-B’’) Confocal images of embryos from 16 to 32 hr AEL showing the process of gonad formation. First, PGCs migrate to the middle of the embryo (B). Second, PGCs split into two groups and migrate to both sides of the embryo (B’’). Third, PGCs form two separate gonads (B’’). (C, D) Confocal image of the embryos showing mis-migrated Vasa-positive PGC during germ band extension at 12 hr AEL (C) and during gonad formation at 16 hr AEL (D). Asterisks in C and D mark the mis-migrated PGCs with Vasa (Green) and DNA (White) staining. Scale bar indicates 50 µm.

**Additional file 2: Excel Table S1. PGC numbers of different larval stage.**

Counted PGC numbers at different stages of larval development is presented.

**Additional file 3: Figure S2. Distinct morphology of ovary and testis during L2 stage, related to Figure 2.**

(A, B) Confocal images showing distinct structures of L2 testis (A) and ovary (B) with Vasa (Green), α-Spec/Phalloidin (Red) and DNA (White) staining. Yellow dashed lines in B mark somatic cells at both anterior and posterior ends. Scale bar indicates 50 µm.

**Additional file 4: Figure S3. Phalloidin, α-Spec, Edu and Cleaved-Caspase3 staining in larval ovaries, related to Figure 3.**

(A) Confocal image of L3 ovary with Vasa (Green), Phalloidin (Red), α-Spec (Blue) and DNA (White) staining. Phalloidin staining largely overlaps with α-Spec staining. However, Phalloidin but not α-Spec labels ring canal (indicated by dotted circle). (B) Confocal images of control and α-Spec-RNAi L2 ovaries. Compared with control ovary, α-Spec-RNAi ovary shows little α-Spec straining. (C) Confocal image of Edu-treated L3 ovary with Vasa (Green), Edu (Red), α-Spec (White) and DNA (Blue) staining. Edu does not label one PGC cyst (dashed outline) connected by α-Spec labelled fusome. (D) Confocal image of L3 ovary of with 6 d ovary with Vasa (Green), Phalloidin (Red), Cleaved-Caspase3 (White) and DNA (Blue) staining showing an apoptotic cyst (dashed outline). All scale bars indicate 50 µm.

**Additional file 5: Movie S1. 3D reconstruction of L1 gonad.**

3D reconstruction of confocal images of L1 gonad with Vasa (Green) α-Spec/Phalloidin (Red) and DNA (Blue) staining showing 5 fusome-connecting PGC cysts.

**Additional file 6: Movie S2. 3D reconstruction of L2 ovary**

3D reconstruction of confocal images of L2 ovary with Vasa (Green) α-Spec/Phalloidin (Red), DNA (Blue) and PH3 (White) staining showing 2 dividing 4-cell PGC cysts.

**Additional file 7: Figure S4. PGC cyst-like division responds to nutrition status promptly during L2 stage, related to Figure 4.**

(A-E) Confocal images of ovaries of control (A), starved for 2 d (B), 8 hr after refeeding (C), 16 hr after refeeding (D), 24 hr after refeeding (E) L2 ovaries with Vasa (Green), α-Spec/Phalloidin (Red), DNA (Blue) and PH3 (White) staining. (A) one well-fed control L3 ovary with PH3+ PGC cyst. (B) one L2 ovary 2 d after starvation showing no PH3+ PGC cyst. (C) one starved L2 ovary after 8 hr refeeding showing no PH3+ PGC cyst. (D-E) PH3+ PGC cyst are detected in starved L2 ovary at 16 hr (D) and 24 hr (E) after refeeding. (F) Quantification of percentages of ovaries with PH3-postive PGCs from three to five pooled independent replicates for (A-E) using Fisher’s Exact Test. (G and H) Confocal images of mock-treated (G) and Rapamycin-treated L2 ovaries for 16 hr (H) with Vasa (Green), α-Spec/Phalloidin (Red), DNA (Blue) and PH3 (White) staining. (I) Quantification of percentages of ovaries with PH3-postive PGCs from three pooled independent replicates for (G-H) using Fisher’s Exact Test. The number of samples (n) in each group is shown above the X axis for F and I. *** p<0.001,** p<0.01,*p<0.05, ns indicates not significant. The dash outlines mark the PH3+ PGC cysts. Scale bar indicates 50 µm (for A-E, G and H).

**Additional file 8: Figure S5. Cross sections of late wL4 ovary, related to Figure 5.**

(A) Confocal microscopy image and cross sections of late wL4 ovary showing the pre-ovariole and oviduct. Vasa (Green), α-Spec/Phalloidin (Red), PH3 (Blue) and NDA (White). Scale bar indicates 50 µm.

**Additional file 9: Figure S6. 20E does not trigger morphological change in L2 or L3 ovaries, related to Figure 6.**

(A-D) Confocal images of ovaries in L2 (A and B) or L3 (C and D) with mocked treatment (A and C) and 20E treatment (B and D). (A and B) L2 Mocked-treated and 20E-treated ovaries show normal structure. (C and D) L3 Mocked-treated and 20E-treated ovaries show normal structure. Vasa (Green), α-Spec/Phalloidin (Red), DNA (White). Scale bar indicates 50 µm.
